# Cerebral large artery occlusion in chronic graft-versus-host disease: A case report

**DOI:** 10.1097/MD.0000000000028263

**Published:** 2021-12-23

**Authors:** Ying Li, Feng Gao, Wei Sun, Zhaoxia Wang, Haiqiang Jin

**Affiliations:** Department of Neurology, Peking University First Hospital, Beijing, China.

**Keywords:** central nervous system, graft-versus-host disease, large vessel occlusion, vasculitis

## Abstract

**Rationale:**

Cerebral large artery occlusion in chronic central nervous system graft-versus-host disease after allogeneic hematopoietic stem cell transplantation (allo-HSCT) was very scarce. We described a young patient with bilateral white matter lesions and symptomatic internal carotid artery occlusion after allo-HSCT with the history of aplastic anemia.

**Patient concerns:**

A 17-year-old girl with the history of aplastic anemia developed recurrent headache and sudden hemiplegia of right limbs 2 years after allo-HSCT.

**Diagnoses:**

She was diagnosed with skin chronic graft-versus-host disease 19 months after allo-HSCT. Brain magnetic resonance imaging showed bilateral subcortical white matter abnormal signals and hyperintensity of left fronto-parietal lobe on diffusion weighted imaging and corresponding hypointense apparent diffusion coefficients indicating acute infarction. CT angiography revealed thrombosis in left internal carotid artery. Carotid plaque high-resolution magnetic resonance imaging showed annular enhancement of vascular wall revealing signs of vasculitis.

**Interventions:**

Intravenous immunoglobulin, methylprednisolone, and anticoagulant therapy were used to treat the patient.

**Outcomes:**

The patient's symptoms gradually resolved and she could walk with assistance after 3 weeks before returned home.

**Lessons:**

Chronic graft-versus-host disease-associated vasculitis could involve cerebral large vessels which warrants further study.

## 1 Introduction

Chronic graft-versus-host disease (GVHD) associated with allogeneic hematopoietic stem cell transplantation (allo-HSCT) is a complicated process mediated by engrafted, immunocompetent donor T cells against host histocompatibility antigens, which most commonly involves the skin, gastrointestinal tract, and liver.^[1,2]^ Chronic GVHD rarely affects the central nervous system (CNS) that may present with cerebrovascular disease, CNS demyelinating disease, and immune-mediated encephalitis.^[1]^ Cerebral vasculitis involving large vessels after GVHD is a rare complication and only 3 cases have been reported to date.^[3–5]^ We report a case of vasculitis-like syndrome associated with GVHD presenting with white matter lesions and internal carotid artery occlusion.

## 2 Case presentation

**Figure 1:**
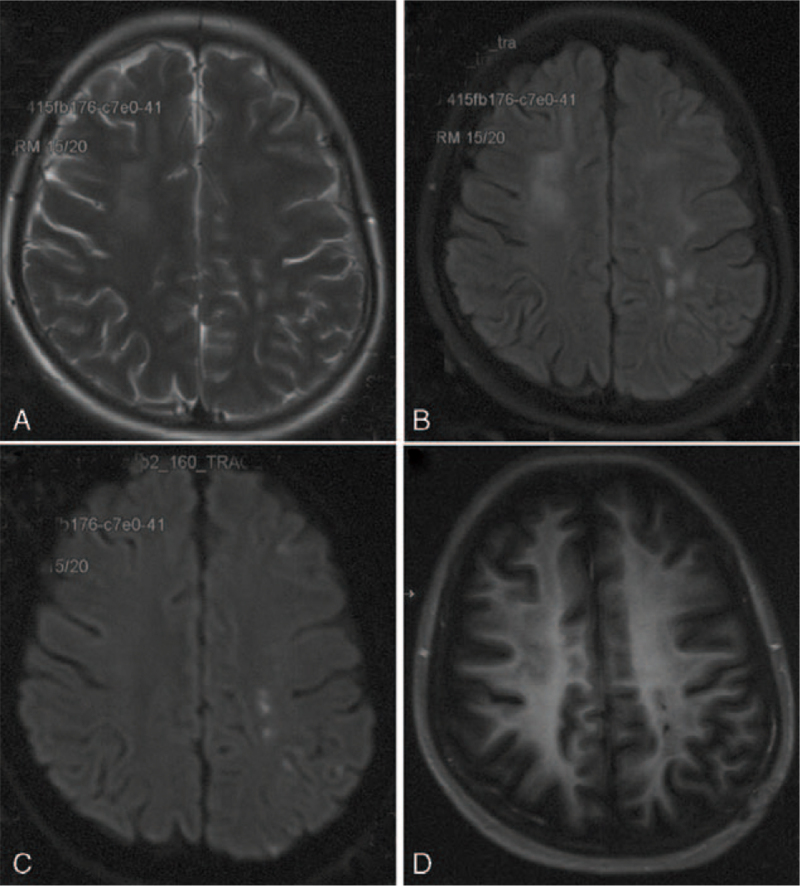
Brain MRI before admitted revealed bilateral fronto-parietal subcortical white matter abnormal signal with slightly higher T2WI and FLAIR signal and hyperintense of left fronto-parietal lobe on DWI (A–C). No enhancement was found on brain enhanced MRI after admission (D). FLAIR = fluid attenuated inversion recovery, DWI = diffusion weighted imaging; MRI = magnetic resonance imaging, T2WI = T2 weighted image.

**Figure 2:**
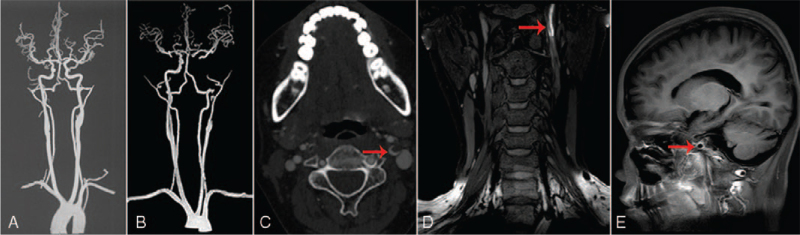
CT angiography 9 months before admitted showed normal extracranial and intracranial vessels (A). CT angiography after admitted revealed occlusion in left internal carotid artery and a crescentic shape of real lumen (B and C). Carotid plaque high-resolution MRI showed annular enhancement of vascular wall (D and E). MRI = magnetic resonance imaging.

A 17-year-old girl with severe aplastic anemia received allo-HSCT with mother donor in 2018. Unfortunately, skin chronic GVHD occurred 19 months after transplantation and the symptoms were relieved after the treatment with tacrolimus, corticosteroids, and ruxolitinib as anti-rejection drugs. The patient fell down frequently because of both lower limbs weakness and developed recurrent headache 2 years after transplantation. Computed tomography angiography (CTA) revealed no abnormal finding 9 months before admission in another hospital (Fig. 2A). The patient developed sudden paraesthesia and hemiplegia of right limbs and dysarthria as well as transient vision loss of left eye after stopping corticosteroids for 3 days (20 days before admitted). Neurological examination revealed tetraparesis and hypertonia of lower limbs requiring wheelchair, scissors gait and positive bilateral Babinski sign. Brain magnetic resonance imaging (MRI) showed bilateral fronto-parietal subcortical white matter abnormal signals with slightly lower T1WI signal, slightly higher T2 weighted image, and fluid attenuated inversion recovery signal (Fig. 1A and B) and hyperintense of left fronto-parietal lobe on diffusion weighted imaging (Fig. 1C) and corresponding hypointense apparent diffusion coefficients indicating acute infarction. Brain enhanced MRI revealed thrombosis in left internal carotid artery without enhancement of bilateral white matter lesions (Fig. 1D). CTA and carotid plaque high-resolution MRI showed occlusion of left internal carotid artery and annular enhancement of vascular wall (Fig. 2B, D, and E), and real lumen had a crescentic shape (Fig. 2C) which is different from crescentic intramural hematoma of carotid-artery dissection. Carotid ultrasonography showed occlusion of left internal carotid artery without intramural hematoma and intimal flap.

Cerebrospinal fluid (CSF) analysis showed normal protein, glucose, and white blood cell and was negative for bacterial, viral or fungal infections. Autoimmune encephalitis markers of both serum and CSF were negative. She had oligoclonal bands in CSF (type II). Neuromyelitis optica and myelin oligodendrocyte glycoprotein antibodies were negative. Antinuclear antibodies titers were 1:100. Other autoantibodies for connective tissue diseases, lupus anticoagulant, anticardiolipin antibody, anti-β2-glycoprotein antibody, antineutrophil cytoplasmic antibody, protein C, protein S, erythrocyte sedimentation rate, and C reactive protein were normal or negative. MRI of cervical vertebrae, thoracic vertebrae and lumbar vertebrae showed no abnormality. Also there were no positive findings of microembolus detection, contrast-enhanced transcranial Doppler ultrasound and ultrasonic cardiogram.

In the absence of relapse of aplastic anemia and ruling out other possible causes, we considered that vasculitis involving large and small vessels associated with CNS-GVHD may be the cause of internal carotid artery occlusion and intracranial white matter lesions. The patient was treated with intravenous immunoglobulin (0.4 g/kg for 5 days) and methylprednisolone (40 mg daily for 5 days) followed by tapering doses of oral prednisolone and rivaroxaban (15 mg daily) therapy. The patient's symptoms gradually resolved and she could walk with assistance after 3 weeks before returned home. The patient's neurological condition was stable 3 months after discharge.

## 3 Discussion

Chronic GVHD occurs in 30% to 50% of transplant patients from related donors,^[3]^ but neurological complications are rare and 47 cases of CNS-GVHD have been reported so far which presented with stroke-like episodes or lacunar syndrome, acute disseminated encephalomyelitis or multiple sclerosis like presentation, encephalopathy or encephalitis and other atypical symptoms.^[6]^

Openshaw described six diagnostic criteria for chronic CNS-GVHD in 2009.^[7,8]^ Although our patient has dissemination in space and dissemination in time which met the diagnostic criteria of multiple sclerosis, the morphology of intracranial lesions was inconsistent with that of typical multiple sclerosis. In addition, the patient's previous skin chronic GVHD suggested that the CNS manifestations and lesions might be explained by the same reason. Furthermore, oligoclonal bands and the improvement of symptoms after immunosuppressive treatment supported the diagnosis of CNS-GVHD. Li et al^[6]^ found that lymphocytic vasculitis has a prominent position in the existing CNS-GVHD histological data, so we speculated that the white matter lesions of the patient was caused by vasculitis involving small vessels associated with CNS-GVHD after excluding other causes.

The precise cause for this patient's subacute artery occlusion within 9 months is not known. The young patient lacked of common risk factors for cerebrovascular disease, and vascular examination showed no evidence of atherosclerotic plaque in cerebral vessels and other vessels. She also denied the history of neck trauma and pain, CTA and carotid plaque high-resolution MRI revealed that real lumen had a crescentic shape which is different from crescentic intramural hematoma of carotid-artery dissection, so evidences of dissection were insufficient. As for cerebral vasculitis, the patient did not have any other manifestations of infection and microbiological examinations of blood and CSF were negative, so infectious etiologies could be excluded. Vasculitis secondary to autoimmune diseases was also excluded by multiple serum markers. Owing to the history of skin chronic GVHD and coexistent white matter lesions caused by possible GVHD-associated vasculitis, we suspected that the most likely cause of artery occlusion is vasculitis in association with GVHD.

Stroke is a common manifestation of CNS-GVHD, but macroangiopathy is rarely observed. Only 3 stroke patients with macroangiopathy after allo-HSCT have been reported, and all of them were vasculitis. Campbell et al^[3]^ reported a patient of cerebral hemorrhage after GVHD and angiography showed multiple segments of abnormally dilated branches of the arteries suggesting cerebral vasculitis of large vessels. Padovan et al^[4]^ described multifocal distribution of inflammatory infiltrations of blood vessel walls and perivascular areas in a patient with multiple cerebral hemorrhage. Nakayama et al^[5]^ demonstrated that an ischemic stroke patient with middle cerebral artery occlusion owing to noninfectious cerebral vasculitis associated with GVHD was treated with superficial temporal artery-middle cerebral artery bypass and histological and immunohistochemical examinations of the affected vessel revealed inflammatory cell infiltrations of perivascular areas.

The mechanism of cerebral vasculitis caused by GVHD remains unclear. Previous cases with biopsy results revealed CD3, CD4, and CD8 T cells infiltration of perivascular areas.^[6]^ An experimental mouse model demonstrated parenchymal lymphocytic inflammation, microglia activation, and mild cerebral angiitis after allo-HSCT.^[9]^ Therefore, inflammatory cells infiltration owing to chronic GVHD may promote arterial remodeling and pathological characteristics could be crucial for the diagnose of cerebral vasculitis.^[5,10]^ Unfortunately, the patient's biopsy was not performed.

Our patient had both white matter lesions and internal carotid artery occlusion which probably caused by GVHD-associated vasculitis involving large and small vessels. As diagnostic markers specific for GVHD have not been identified yet, the definite relationship between GVHD and vasculitis still remains to be established and secondary vasculitis have to be excluded.

## Author contributions

**Conceptualization:** Ying Li, Haiqiang Jin.

**Data curation:** Ying Li, Haiqiang Jin.

**Formal analysis:** Ying Li.

**Supervision:** Feng Gao, Wei Sun, Zhaoxia Wang, Haiqiang Jin.

**Writing – original draft:** Ying Li.

**Writing – review & editing:** Feng Gao, Wei Sun, Zhaoxia Wang, Haiqiang Jin.
